# NFTest: automated testing of Nextflow pipelines

**DOI:** 10.1093/bioinformatics/btae081

**Published:** 2024-02-10

**Authors:** Yash Patel, Chenghao Zhu, Takafumi N Yamaguchi, Yuan Zhe Bugh, Mao Tian, Aaron Holmes, Sorel T Fitz-Gibbon, Paul C Boutros

**Affiliations:** Jonsson Comprehensive Cancer Center, University of California, Los Angeles, Los Angeles, CA 90095, United States; Institute for Precision Health, University of California, Los Angeles, Los Angeles, CA 90095, United States; Jonsson Comprehensive Cancer Center, University of California, Los Angeles, Los Angeles, CA 90095, United States; Institute for Precision Health, University of California, Los Angeles, Los Angeles, CA 90095, United States; Department of Human Genetics, University of California, Los Angeles, Los Angeles, CA 90095, United States; Jonsson Comprehensive Cancer Center, University of California, Los Angeles, Los Angeles, CA 90095, United States; Institute for Precision Health, University of California, Los Angeles, Los Angeles, CA 90095, United States; Department of Human Genetics, University of California, Los Angeles, Los Angeles, CA 90095, United States; Jonsson Comprehensive Cancer Center, University of California, Los Angeles, Los Angeles, CA 90095, United States; Jonsson Comprehensive Cancer Center, University of California, Los Angeles, Los Angeles, CA 90095, United States; Institute for Precision Health, University of California, Los Angeles, Los Angeles, CA 90095, United States; Department of Human Genetics, University of California, Los Angeles, Los Angeles, CA 90095, United States; Jonsson Comprehensive Cancer Center, University of California, Los Angeles, Los Angeles, CA 90095, United States; Institute for Precision Health, University of California, Los Angeles, Los Angeles, CA 90095, United States; Jonsson Comprehensive Cancer Center, University of California, Los Angeles, Los Angeles, CA 90095, United States; Institute for Precision Health, University of California, Los Angeles, Los Angeles, CA 90095, United States; Department of Human Genetics, University of California, Los Angeles, Los Angeles, CA 90095, United States; Jonsson Comprehensive Cancer Center, University of California, Los Angeles, Los Angeles, CA 90095, United States; Institute for Precision Health, University of California, Los Angeles, Los Angeles, CA 90095, United States; Department of Human Genetics, University of California, Los Angeles, Los Angeles, CA 90095, United States; Department of Urology, University of California, Los Angeles, Los Angeles, CA 90095, United States; Broad Stem Cell Research Center, University of California, Los Angeles, Los Angeles, CA 90095, United States

## Abstract

**Motivation:**

The ongoing expansion in the volume of biomedical data has contributed to a growing complexity in the tools and technologies used in research with an increased reliance on complex workflows written in orchestration languages such as Nextflow to integrate algorithms into processing pipelines. The growing use of workflows involving various tools and algorithms has led to increased scrutiny of software development practices to avoid errors in individual tools and in the connections between them.

**Results:**

To facilitate test-driven development of Nextflow pipelines, we created NFTest, a framework for automated pipeline testing and validation with customizability options for Nextflow features. It is open-source, easy to initialize and use, and customizable to allow for testing of complex workflows with test success configurable through a broad range of assertions. NFTest simplifies the testing burden on developers by automating tests once defined and providing a flexible interface for running tests to validate workflows. This reduces the barrier to rigorous biomedical workflow testing and paves the way toward reducing computational errors in biomedicine.

**Availability and implementation:**

NFTest is an open-source Python framework under the GPLv2 license and is freely available at https://github.com/uclahs-cds/tool-NFTest. The call-sSNV Nextflow pipeline is available at: https://github.com/uclahs-cds/pipeline-call-sSNV.

## 1 Introduction

With the advent of high-throughput technologies ranging from, e.g. high-throughput sequencing to live-cell imaging and mHealth, biomedical research has seen a sharp increase in the speed and affordability of generating large datasets. The resulting ongoing expansion in data volume has been paralleled by a growing complexity in the tools and technologies used in research. Biomedical discoveries often involve stitching together complex workflows of established and novel algorithms to sequentially process data into more refined forms. With the increased complexity, significant amounts of time and effort are drawn into the development and maintenance of these workflows (Dash *et al.* 2019, Cremin *et al.* 2022), which are often called “pipelines.”

Pipelines are often implemented through the use of workflow orchestration frameworks built for data processing. These frameworks aim to minimize manual control of data flow from step to step and enable scalability and reproducibility of analyses with support for cluster and cloud computing environments. Widely used orchestration frameworks in computational biology include Common Workflow Language, Snakemake, Galaxy, and Nextflow (Köster and Rahmann 2012, Di Tommaso *et al.* 2017, Crusoe *et al.* 2022, The Galaxy Community 2022).

This growing use of complex workflows in biomedicine has placed a heavy burden on software development practices. Errors in any single component of the workflow, or in the way the individual components are stitched together, can dramatically change the final outputs. This can lead to wasted compute time, loss of precision or even spurious results. Novel development and a focus on producing a functional set of tools often takes precedence over systematic and automated testing. Small changes to workflows can be missed and undocumented, hindering reproducibility and reducing accuracy (Baresi and Pezzè 2006). To avoid these errors, software engineering best practices are becoming wide-spread in biomedical data processing. These include standardized linting (Louridas 2006), input/output standardization (Silva *et al.* 2017), and test-driven development (Janzen and Saiedian 2005, Patel *et al.* 2024).

The last of these is particularly critical, but also burdensome computationally and for developers. In the early phases of workflow innovation, testing is often performed manually. Even in later stages, when some automation is often present, frequent updates to individual tools can challenge the capacity of teams to reliably test their workflows. For example, Picard, a set of command line tools for manipulating high-throughput sequencing data, and the Genome Analysis Toolkit, a set of tools for genome analysis, are updated and enhanced with new releases every couple of months. PLINK, used for computationally efficient whole-genome association analysis, goes through update cycles shorter than 1 month (Purcell *et al.* 2007, McKenna *et al.* 2010, Broad Institute 2019). The lack of generalized testing frameworks for common workflow orchestration languages often results in researchers using simple scripts in Perl, Python or Bash that are difficult to adapt to new workflows, or to modify to changing needs. As a result, workflow testing becomes even more limited in scope.

Nextflow is a rapidly emerging and broadly supported workflow orchestration framework, with implementation and deployment support for complex parallel and reactive workflows on high-performance clusters and clouds, and includes support for software containers and adaptability of common scripting languages. Thus, Nextflow facilitates scalable and data-driven scientific workflows. To automate workflow testing and validation in Nextflow, we created NFTest. NFTest supports a wide range of Nextflow features and includes customizable validation options for pipeline results. It reduces the barrier to workflow testing, and provides a path toward reducing the rate of computational errors in biomedicine.

## 2 Results

NFTest is a light-weight, customizable and easy-to-use method for automating testing of Nextflow workflows. With Nextflow offering a wide range of features such as multiple config files, configuration profiles, and parameter files, pipelines often become complex. NFTest includes support for these features in the definition of tests and enables automation once test cases are defined, so developers no longer need to manually set up testing with all pipeline features for every development cycle. NFTest automates both test setup through an initialization functionality and test execution comprising configuration and parameter handling, workflow execution, and output validation. Through implementation of multiple comparisons and assertion methods, NFTest also provides a robust way to test a pipeline by making different assertions such as pipeline completion, MD5 checksum (with planned support for other checksum formats such as SHA512) comparison between test and expected output files, and custom scripts to define comparisons between files. It features a simple command line interface through which workflow testing can be implemented in three steps: initialize the testing framework, define parameters and test cases, and execute the tests.

The first step of NFTest is to initialize the test framework. This can be achieved simply by calling nftest init. With this call, NFTest will automatically set up the basic testing directory structure along with generating a template through which test cases can be defined. For initialization, NFTest will create the directory for holding test files and copy templates of a global configuration file and a YAML containing the structure for defining test cases. These files can then be expanded and populated to define the necessary set of test cases for the pipeline being tested.

The second step of NFTest is to define the parameters and test cases. Test cases can be defined through modification of the template generated during initialization to indicate the settings, such as Nextflow script, configuration file(s), configuration profile(s), and assertions, for each test case and the Nextflow script to be tested. NFTest supports robust definition for the workflow to be tested through the script, which can run individual processes from the pipeline, individual workflows from the pipeline, and the entire pipeline. Parameters can be defined through environment variables to configure options such as output and working directory without requiring explicit updates to the test cases on a per-developer basis while still reconciling settings to automatically identify output files for comparison and validation. Validation methods are also variable and customizable. The methods include options for no assertions for test cases intended to test successful pipeline completion, checksum assertions with md5, and custom script assertions that accept any script taking in two files (the actual and expected files) as arguments and returning a pass or fail to indicate the comparison status between the input files.

The third step of NFTest is to execute the tests as development progresses. With test cases defined, developers can execute the tests by defining user-specific parameters through environment variables and simply calling nftest run to automatically detect the test cases and perform the testing for all enabled cases.

**Figure 1.**
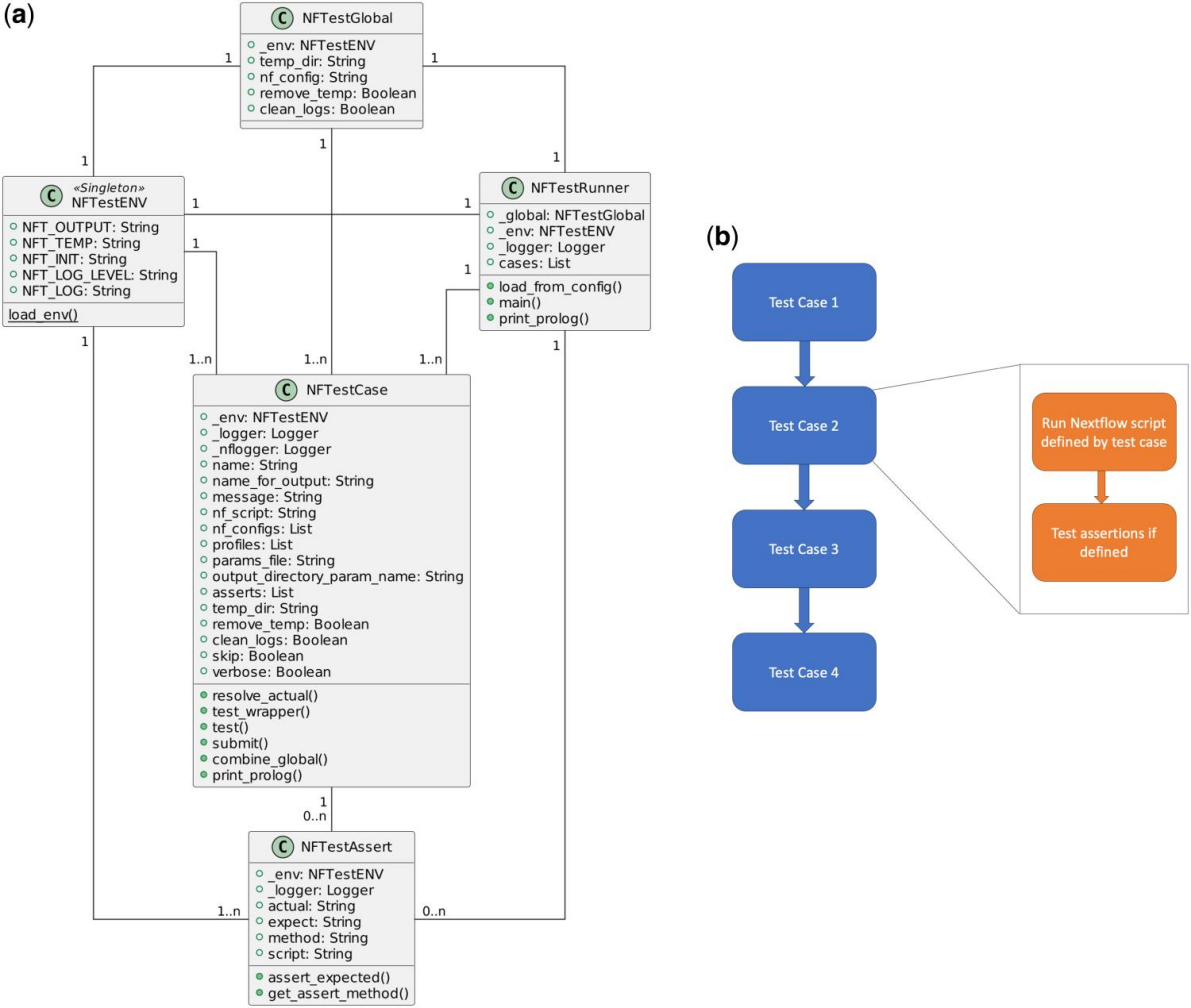
Architecture and workflow of testing. (a) Class diagram indicating relationships between environment variables, global settings, and case-specific settings. (b) Workflow of execution of test cases, indicating where the scripts are run and where assertions are made.

With a focus on facilitating testing of complex workflows that incorporate a wide range of Nextflow features, NFTest is designed in a hierarchical structure with test cases defined using two levels: global and case-specific (Fig. 1). The global level defines the default settings for the Nextflow temporary directory, global Nextflow configuration, and test output cleanup options. The case-specific level defines a set of test cases with support for additional settings that can override the global settings as necessary. Nextflow provides a range of options, including the ability to run a pipeline directly from a GitHub repository, multiple ways to define parameters (config files and params-files), the possibility to provide multiple config files, and profiles to bundle enablement/disablement of sets of configurations. NFTest provides support for defining tests including any combination of these options. With complex workflows, a series of tests are often necessary to cover various use-cases and settings. To facilitate this, NFTest provides the option to enable and disable certain test cases as default and use the command line interface to run test cases that may be disabled as default without having to modify the test definitions. The command to run a specific subset of test cases, regardless of whether the cases are enabled as default or not, is simply nftest run “test case 1” “test case 2” “test case 6.”

We demonstrate the ease of testing and development with NFTest through implementation with two independent Nextflow workflows: nf-core’s sarek pipeline for germline and somatic variant detection from next-generation sequencing data and a novel pipeline yielding integrated results from four different tools for somatic single nucleotide variant (sSNV) detection with a workflow for intersecting the resulting variant call sets (Garcia *et al.* 2020). nf-core’s sarek pipeline includes configurations using Nextflow profiles and specific settings for indicating output directories for any generated files. NFTest includes functionality to specify the organization and repository as the main script for testing, along with support for defining test cases using specific profiles and output directory parameter names. As a demonstration, we defined a test of sarek using the test and docker profiles with specific assertions for variant calls resulting from Strelka produced by the workflow. We show that developers can define custom assertions between files using scripts. The sarek test makes use of human chromosome 21 as a test sample and performs variant calling with Strelka2. We implemented a custom script to assert a match between only the variant call sets in the output files and not the headers of the files.

**Figure 2.**
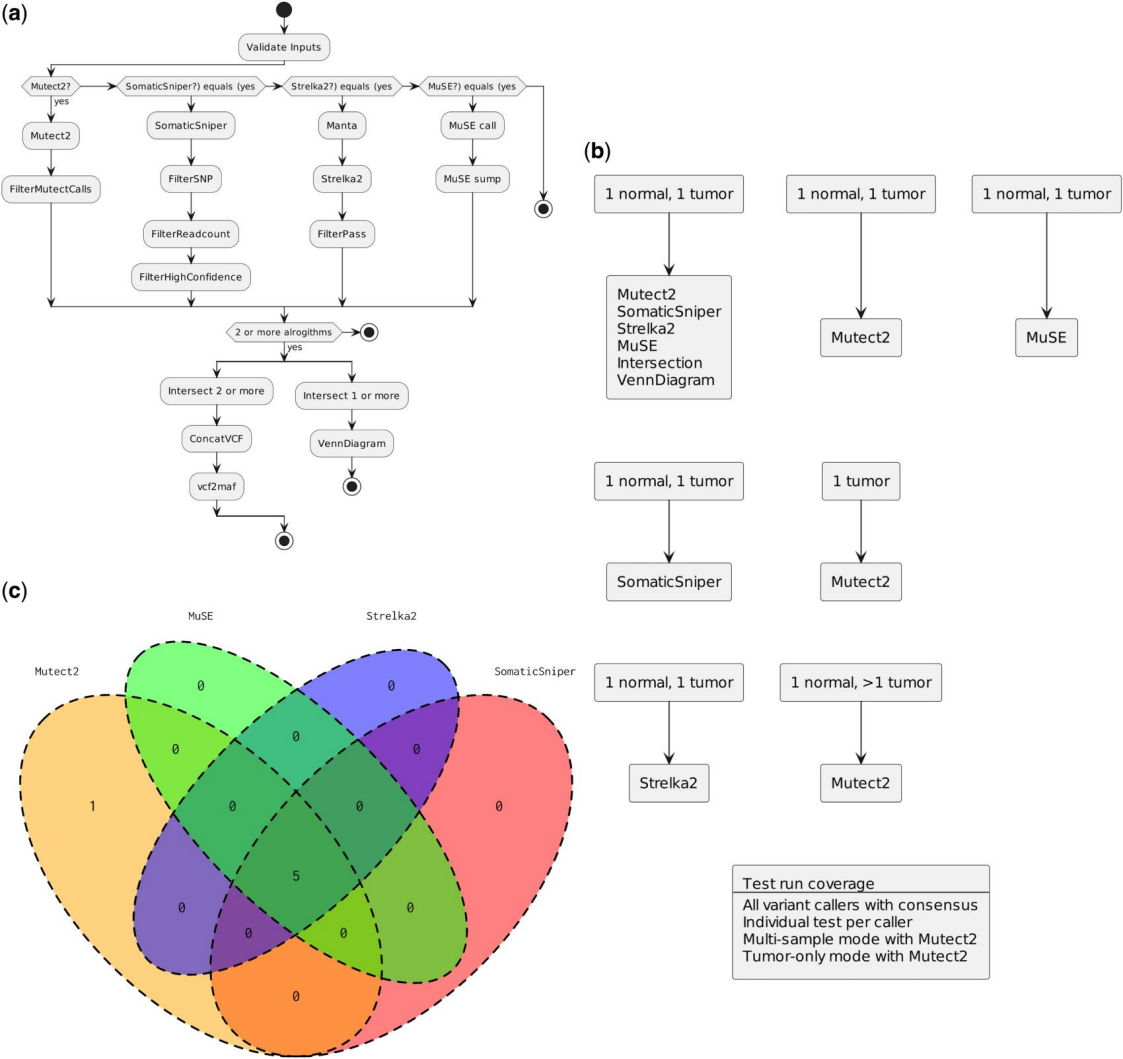
Workflow and testing of somatic SNV calling pipeline. (a) Somatic SNV calling pipeline flowchart through Mutect2, SomaticSniper, Strelka2, and MuSE algorithms culminating in intersection workflow. (b) Set of tests implemented for call-sSNV pipeline covering different inputs and algorithms. (c) Example intersection diagram of consensus variants; assertions are made to ensure the same diagram is produced through NFTest.

We also implemented NFTest with a novel pipeline for sSNV calling. The call-sSNV pipeline, implemented in Nextflow, supports four SNV calling tools: Mutect2, SomaticSniper, Strelka2, and MuSE (McKenna *et al.* 2010, Larson *et al.* 2012, Fan *et al.* 2016, Kim *et al.* 2018). The pipeline accepts a set of normal and tumor Binary Sequence Alignment/Map files and generates somatic variant call sets through each of the four tools (Li *et al.* 2009, Danecek *et al.* 2011). As a final step, the pipeline also implements a workflow to intersect the call sets from the different callers using BCFtools (Danecek *et al.* 2021). With options for specifying combinations of tools and the different tools accepting different inputs, call-sSNV calls for a need for several test cases to ensure proper function. We implemented NFTest with call-sSNV (Fig. 2) to capture these cases with VCF comparisons done on variant calls from each tool and on the intersected variant calls along with the associated Venn Diagram: a test with all tools, a test for each individual caller, a test for Mutect2 tumor-only mode, and a test for Mutect2 multiple sample mode (Chen and Boutros 2011). Each of these tests demonstrates the end-to-end testing functionality of NFTest, with test success determined by pipeline success. A subsampled tumor dataset, comprising ∼0.01% of the genome, from the simulated tumor data generated through the SMC-Het challenge was used as the test dataset (Salcedo *et al.* 2020). The test input and output files are available with the latest release of call-sSNV here: https://github.com/uclahs-cds/pipeline-call-sSNV/releases/download/v8.0.0-rc.1/test_files.tar.gz.

Through testing with these data on a system with 16 CPUs and 32 GB of RAM, we also demonstrate the light resource requirements of NFTest. We ran 10 benchmarking runs of call-sSNV on the 0.01% of genome simulated tumor data from the SMC-Het challenge through NFTest and 10 runs without NFTest, while monitoring the percent CPU usage, percent RAM usage, and disk space usage. For CPU usage, call-sSNV alone peaked at median (IQR) 84.78% (83.84–84.92), while call-sSNV through NFTest peaked at 85.68% (85.32–86.09). For memory usage, call-sSNV alone peaked at median (IQR) 36.45% (33.73–37.44), while call-sSNV through NFTest peaked at 34.75% (34.27–37.02). With respect to runtime, on the test system, call-sSNV alone runtime was 14 min 37 s (13 min 49 s to 14 min 58 s), while call-sSNV with NFTest runtime was 14 min 52 s (14 min 3 s to 15 min 22 s). As a whole, testing added ∼0.9% CPU overhead, no memory overhead (all values were within the run-to-run stochasticity of memory usage), and approximately 15 s (or 1.7%) to total run time. For the simulated data used for testing call-sSNV, the intermediate disk usage peaked at 250 MB with the final outputs using 150 MB. The simulated test files used for testing call-sSNV require 33 MB, while the reference files require 30 GB. The Docker containers, once pulled from the container registries, require 12 GB of disk space. Testing results in no effect on output file size, aside from a tiny log file in the single-digit MB range.

In addition to NFTest, nf-test is another open-source testing framework for Nextflow. Both NFTest and nf-test allow for execution and testing of Nextflow workflows, processes, and functions, for assertions to be made on output files, and for generating testing code templates. Both frameworks additionally include support for snapshot testing by comparing test runs with existing snapshot test runs. NFTest provides support for tests using configuration files and Nextflow profiles and allows for customizable comparison scripts that can leverage various environments and programming languages. Though nf-test is not published as far as we know, it is an open-source tool like NFTest and, as such, similarities in features may accumulate as both aim to provide a testing framework for Nextflow. NFTest will continue to be expanded with additional features such as validating outputs directly written to a database or cloud storage containers.

Reproducibility, and therefore testing and validation, are key for complex bioinformatics workflows. Workflow testing is difficult given the sheer scale and intricacy of data and the processing pipelines along with a priority set on developing functioning workflows. By minimizing developer programming effort and providing a customizable testing framework, NFTest paves the way toward easy testing, maintenance, and development of complex bioinformatics workflows with a range of testing assertions and feature customizability while minimizing extensive programming and maintenance effort from developers.

## Data Availability

No new data were generated or analysed in support of this research.
